# Effects of static stretching and walking during inter-set intervals of resistance training on muscle fatigue of the quadriceps

**DOI:** 10.3389/fspor.2024.1483972

**Published:** 2025-01-15

**Authors:** Kosuke Takeuchi, Hiroaki Inoue, Motoka Fujiwara, Taiki Shimizu, Chiharu Nagai, Kosei Mizuno, Masatoshi Nakamura

**Affiliations:** ^1^ Department of Physical Therapy, Faculty of Rehabilitation, Kobe International University, Kobe-shi, Japan; ^2^ Department of Physical Therapy, Faculty of Rehabilitation Sciences, Nishi Kyushu University, Kanzaki-cho, Japan

**Keywords:** resistance training, total amount of volume load, exhaustion, recovery, active rest

## Abstract

**Introduction:**

Inter-set rest intervals are essential to reduce muscle fatigue and increase the total amount of volume of resistance training. Static stretching and walking may increase muscle blood flow and promote recovery during inter-set rest intervals. Therefore, the purpose of this study was to investigate the effects of 20 seconds of static stretching and walking during inter-set rest intervals of leg extension exercises on the number of repetitions to exhaustion, total amount of volume, and flexibility of the quadriceps (joint range of motion and muscle hardness) in untrained healthy males.

**Methods:**

Fourteen healthy, untrained men performed three different interventions (passive rest, static stretching, and walking) during inter-set rest intervals of a leg extension exercise with a 70% load of maximum muscle strength, in random order. The range of motion and muscle hardness of the quadriceps were measured before and immediately after all interventions using a goniometer and a portable muscle hardness meter. The number of repetitions to exhaustion and total amount of volume load (load × repetitions) of the leg extension exercise were assessed. Repeated two-way ANOVA and a Friedman test were used to analyze the variables.

**Results:**

Range of motion and muscle hardness were increased after all interventions (both *p* < 0.05). There was no significant difference in the number of repetitions to exhaustion (*p* = 0.651) or total amount of volume load (*p* = 0.206) between interventions.

**Discussion:**

These results indicated that static stretching and walking during inter-set rest intervals did not influence the change in flexibility and muscle fatigue of the leg extension exercise.

## 1 Introduction

Resistance training is widely used to improve the performance of athletes and the well-being of the general population (1, 2). Total amount of volume load is one of the essential factors for the effectiveness of resistance training (3–5). Total amount of volume load is calculated from the loads used in the training and the number of repetitions (6–8). Therefore, reducing muscle fatigue during resistance training is necessary, thereby maintaining repetitions at high loads and increasing total amount of volume load.

Inter-set rest intervals are essential to reduce muscle fatigue and increase the total amount of volume load of resistance training (3, 5). Rest intervals should be long enough to replenish adenosine triphosphate and phosphocreatine and to remove accumulated lactic acid (5, 9). If the recovery during the rest intervals is insufficient, muscle fatigue might compromise the ability to sustain repeated high-force muscular contractions because of the increment in reliance on glycolytic energy production (9).

A previous systematic review study indicated a similar effect of short and long inter-set rest intervals for gains in muscle hypertrophy (3). On the other hand, previous studies reported greater muscle hypertrophy from resistance training with longer rest intervals (3 min) compared to shorter rest intervals (60 s) because of the greater total amount of volume load in longer rest intervals (9–11). However, it was reported that the most common reason for not performing resistance training is a lack of time (12). Therefore, it is necessary to establish a rest interval method that can effectively reduce muscle fatigue in a short time to increase adherence to resistance training for individuals who do not have time for the training.

Static stretching (13) and aerobic exercise (14) acutely increase muscle blood flow, and this increased flow may reduce lower limb muscle fatigue by eliminating the accumulation of metabolites such as lactate. Lopes et al. (13) reported that static stretching for lower muscles (hip extensors, hip adductors, quadriceps, and hamstrings) before high-intensity aerobic exercise on a cycling ergometer reduced the accumulation of lactic acid. On the other hand, static stretching decreases muscle strength by decreasing muscle activity of plantar flexors (15, 16). Stretching-induced changes in muscle neural activation impair cycling efficiency, resulting in decreased time to exhaustion (13). Stretching-induced muscle strength deficit occurs after more than 30 or 45 s of static stretching (17, 18). Therefore, static stretching for less than 30 s during an inter-set rest interval may reduce muscle fatigue without compromising performance and may increase the number of repetitions to exhaustion of resistance training. However, to the best of our knowledge, the effects of static stretching and walking during the inter-set rest intervals of resistance training on the number of repetitions to exhaustion have not been investigated.

The passive properties of the muscle are often measured by using muscle hardness (19–22) and stiffness (23). Muscle hardness is the resistance value when a muscle is pressed in a minor axis, and stiffness is the resistance value when a muscle is stretched in the major axis. Previous studies showed that static stretching changed the passive properties of the muscle, but resistance training and aerobic exercise did not, although these interventions could increase the range of motion (ROM) (23). Change in ROM is attributed to the stretch tolerance and passive properties of the muscle (23, 24) and previous studies suggested that resistance training and aerobic exercise increase ROM due to an increase in stretch tolerance. However, the immediate effects of static stretching and walking during inter-set rest intervals are not clear.

Therefore, the purpose of this study was to investigate the effects of 20 s of static stretching or walking during inter-set rest intervals of leg extension exercises on the number of repetitions to exhaustion, total amount of volume load, and quadriceps flexibility [joint range of motion (ROM) and muscle hardness] in untrained healthy males. The hypothesis of this study was that static stretching and walking intervention during inter-set rest intervals would increase the number of repetitions to exhaustion and the total amount of volume load of leg extension exercises.

## 2 Materials and methods

### 2.1 Experimental protocol

**Figure 1:**
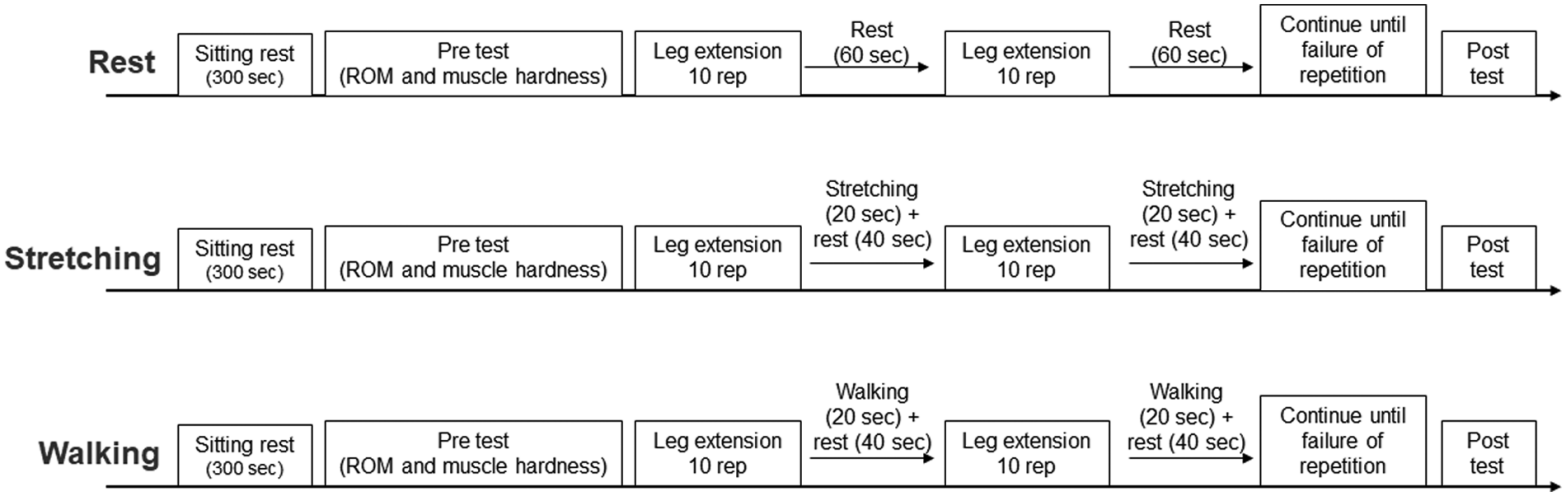
Experimental protocol. ROM: range of motion, rep: repetitions.

In the present study, a randomized cross over design was used. The participants visited four times with an interval of ≥2 weeks between visits. On the first day, the maximal muscle strength of the knee extension was measured, and intervention methods were practiced. In addition, ROM and muscle hardness were measured twice to assess the intraclass correlation coefficient (ICC). In the second through fourth visits, the following procedures were used in the experiment: 5 min of sitting rest, pre-ROM test, pre-muscle hardness test, warm-up, knee extension exercise with interventions (passive rest, static stretching, and walking during inter-set rest interval), post-ROM test, and post muscle hardness test (Figure 1). In the warm-up session, the participants practiced leg extension exercises with light loads. To assess the flexibility of the quadriceps in the dominant leg (ball kicking preference) (25, 26), both before and immediately after resistance training, ROM and muscle hardness were measured. The participants determined the dominant leg as the one easier to kick the ball with. In addition, the number of repetitions and total amount of volume load of the knee extension exercises were evaluated. The experiment was performed in a university laboratory, where the temperature was maintained at 25°C.

### 2.2 Participants

Fourteen healthy male university students participated in this study (20.5 ± 0.5 years, 168.9 ± 5.3 cm, 58.6 ± 7.6 kg). Participants were recreationally active but did not participate in resistance training. Participants with a history of pathology in the quadriceps or hamstrings within six months were excluded. The sample size for the number of repetitions to exhaustion was calculated with a study type of one-way ANOVA, power of 80%, alpha error of 0.05, and effect size of 0.52 using G*Power 3.1 software (Heinrich Heine University, Düsseldorf, Germany), and the results showed that the requisite number of participants for this study was 13 participants; thus, 14 participants were recruited to account for possible attrition. The effects of static stretching and walking during the inter-set rest intervals of resistance training on the number of repetitions to exhaustion have not been investigated. Therefore, we calculated the sample size based on Lopes et al. (13), which examined the effects of static stretching before a cycling ergometer test on time to exhaustion. All participants were informed of the requirements and risks associated with their involvement in this study and signed a written informed consent document. The study was performed in accordance with the Declaration of Helsinki (1964). The Ethics Committee of Kobe International University approved the study (Procedure #G2023-188).

### 2.3 Muscle strength measurement

The maximum knee extension strength of one repetition maximum of the dominant leg was measured using a leg extension machine (NR-S; Senoh Corporation, Japan). Ten leg extension exercises were performed with a light load as a warm-up program, followed by a one-minute rest period. Thereafter, the load was increased by 6 kg, and participants performed one leg extension for each weight until they failed to extend. A one-minute rest interval was set between each knee extension. The knee extension was performed, ranging from 0 to 90 degrees of knee flexion. The average value of the muscle strength was 43.7 ± 8.9 kg.

### 2.4 ROM

ROM of the knee extensors was measured in the same fashion as in previous studies (27, 28). Participants were positioned in a kneeling lunge position with a 90-degree flexion of the hip and knee joint of the non-dominant leg and a 30-degree extension of the hip joint of the dominant leg. Thereafter, the knee joint of the dominant limb was passively and slowly flexed to the knee flexion angle just before the participants started to feel discomfort or pain (27–30). ROM of the knee flexion was measured using a goniometer twice, and the average value was used for further analysis.

### 2.5 Muscle hardness

Muscle hardness was measured in the supine position using a portable muscle hardness meter (NEUTONE TMD-N1, Try-All Ltd., Chiba Japan) (19–22). The muscle hardness of the rectus femoris was measured at the midpoint between the anterior superior iliac spine and the proximal end of the patella. The values measured by this device have no units (values were described between 0 and 100). Therefore, based on the manufacturer's report and previous study (22), the values were converted to Newton using the following formula: *N* = 0.0238 × measured value + 0.532. The muscle hardness was measured three times, and the average value was used for the analysis.

### 2.6 Interventions

A unilateral (dominant leg) knee extension exercise was performed. The load of the leg extension was a weight of 70% of the maximum strength. Leg extension exercises were performed with ten repetitions per set with a 60-s rest between sets. Participants grasped the grips with their hands and actively held their bodies during resistance training. The exercise was repeated until muscle fatigue made repetitions impossible, and the number of repetitions was recorded. Leg extensions were performed in a range of 0–90 degrees of knee flexion and were controlled with a metronome to a pace of once every 2 s (concentric and eccentric contractions for 1 s each). During a 60-s rest interval, the following interventions were performed. The total amount of volume load of the leg extension exercise was calculated from load and the number of repetitions.

#### 2.6.1 Passive rest

The participants sat on the leg extension machine for 60 s.

#### 2.6.2 Stretching

Static stretching of the quadriceps was performed for 20 s. The participants were in a single-leg standing position, holding onto the leg extension machine for balance. They grasped the ankle on the dominant side with one hand and moved the leg toward the buttocks, bending the knee as far as possible. Static stretching was performed at the maximum knee flexion angle without pain or discomfort. The remaining 40 s of the interval was resting in a sitting position.

#### 2.6.3 Walking

Participants walked for 20 s at the speed they felt most comfortable. The average walking distance was about 23 m. The remaining 40 s of the interval was resting in a sitting position.

### 2.7 Reliability

The test-retest reliability for both ROM and muscle hardness was determined. On the first day, ROM and muscle hardness were measured twice using the same procedures as those used before and after the intervention. The reliability of ROM (ICC of 0.858) and muscle hardness (ICC of 0.95) were acceptable in this study.

### 2.8 Statistical analyses

The Shapiro–Wilk test was used to assess normal distribution. ROM and muscle hardness data were normally distributed, but the number of repetitions to exhaustion and total amount of volume load data were not normally distributed. For the ROM and muscle hardness data, a repeated two-way ANOVA (time [pre vs. post] × interventions [passive rest vs. stretching vs. walking] was used. For non-normally distributed data (the number of repetitions to exhaustion and total amount of volume load data), a Friedman test was used to analyze the difference between the interventions (passive rest vs. stretching vs. walking). If a significance was detected, *post hoc* analyses using Bonferroni's test were performed. Partial *η*^2^ values were reported to reflect the magnitude of the differences for each treatment (small ≧ 0.01, medium ≧ 0.06, and large ≧ 0.14) (31). Effect sizes were calculated as the mean difference between pre- and post-values divided by the pooled standard deviation. The analyses were performed using SPSS version 25 (SPSS, Inc., Armonk, NY, USA). Differences were considered statistically significant at an alpha of 0.05.

## 3 Results

**Figure 2:**
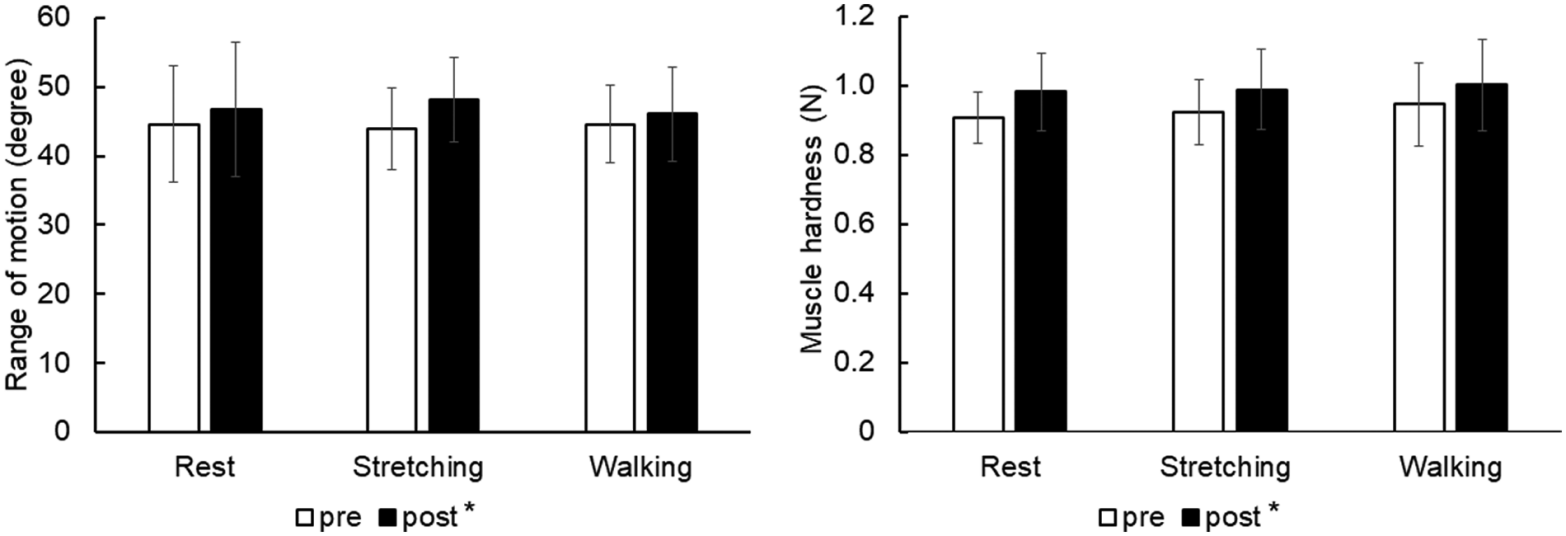
Change in range of motion and muscle stiffness. Values were described as mean ± standard deviation. *p < 0.05 vs. pre.

For ROM, there was no significant interaction (*p* = 0.482, partial *η*^2^ = 0.055) and no main effect for interventions (*p* = 0.925, partial *η*^2^ = 0.006), but there was a significant main effect for time (*p* = 0.015, partial *η*^2^ = 0.376) (Figure 2). It significantly increased after interventions (passive rest, from 44.6 ± 8.4 to 46.8 ± 9.7 degrees, d = 0.78; stretching, from 43.9 ± 5.9 to 48.2 ± 6.1 degrees, d = 0.87; walking, from 44.6 ± 5.7 to 46.1 ± 6.8 degrees, d = 0.67; *p* < 0.01).

For muscle hardness, there was no significant interaction (*p* = 0.733, partial *η*^2^ = 0.024) and no main effect for interventions (*p* = 0.654, partial *η*^2^ = 0.032), but there was a significant main effect for time (*p* < 0.01, partial *η*^2^ = 0.740) (Figure 2). It significantly increased after interventions (passive rest, from 0.91 ± 0.07 to 0.98 ± 0.11 N, d = 1.42; stretching, from 0.92 ± 0.09 to 0.99 ± 0.11 N, d = 1.12; walking, from 0.95 ± 0.12 to 1.00 ± 0.13 N, d = 0.82; *p* < 0.01).

For the number of repetitions to exhaustion (*p* = 0.651) and total amount of volume load (*p* = 0.206) of the leg extension exercises, there was no significant difference between interventions (Table 1).

**Table 1:** The number of repetitions to exhaustion and total amount of volume load.

	Passive rest	Stretching	Walking
Number of repetitions	33 (27–69)	29 (27–61)	29 (28–69)
Total amount of volume load (kg)	1,455 (962–2,440)	1,454 (1,102–2,168)	1,454 (1,127–2,412)

Values were described as median (interquartile range).

## 4 Discussion

The present study examined the effects of static stretching and walking during inter-set rest intervals of resistance training on the number of repetitions to exhaustion, total amount of volume load, and flexibility (range of motion and muscle hardness) of the quadriceps. This study showed an increase in ROM and muscle hardness in all interventions. The number of repetitions to exhaustion and total amount of volume load did not differ between interventions. These results indicated that static stretching and walking during inter-set rest intervals did not positively affect muscle fatigue or flexibility of the leg extension exercises.

The results of this study showed that ROM increased in all interventions. Takeuchi et al. (23) compared the immediate effects of hot water immersion, aerobic exercise, and maximum voluntary contraction on the flexibility and muscle strength of the plantar flexors. They reported that ROM increased after all interventions without any difference between the interventions. In addition, static stretching is also an effective intervention to increase ROM (28, 30, 32). The time course of ROM change during resistance training is not clear. However, the change in ROM with static stretching reaches a plateau approximately 60 s after stretching (33). Therefore, from the results of this study and previous studies (23, 33), it is possible that resistance training significantly increased ROM and that adding static stretching or aerobic exercise to the inter-set rest did not cause further change.

Change in ROM is attributed to changes in the passive properties and stretching tolerance of the muscle (24, 34). Passive properties of the muscle are often evaluated by the parameters of hardness (35) and stiffness (24, 34). Takeuchi et al. (23) reported that aerobic exercise and repeated voluntary maximum muscle contraction increased ROM and stretching tolerance of the plantar flexors but did not change muscle-tendon unit stiffness (23). These data indicated that aerobic exercise and resistance training increased ROM due to a change in stretching tolerance. On the other hand, resistance training causes muscle swelling due to the accumulation of metabolites in the muscle (36, 37). Muscle swelling increases the internal pressure of the muscle and, as a result, increases the muscle hardness, which is the resistance value of a minor axis (37). Static stretching and foam rolling can acutely decrease muscle stiffness and muscle hardness of the quadriceps (35). However, it was reported that 3 min of static stretching is required to decrease muscle stiffness of the rectus femoris (28), and 20 s of static stretching is insufficient time to change the passive properties of the muscle. Previous studies have reported that static stretching for more than 30 s immediately reduces muscle strength (17, 18). On the other hand, static stretching for 20 s does not change muscle strength (32, 38). Therefore, in this study, to avoid the immediate muscle strength deficit caused by static stretching interventions, we used 20-s static stretching, which does not change stiffness, as the intervention. Thus, it was suggested that resistance training itself produced an acute increase in ROM due to increased stretching tolerance, although it increased muscle hardness due to the accumulation of metabolites.

The results of this study showed no significant difference in the number of repetitions to exhaustion or total amount of volume load between all interventions. Insufficient rest intervals during resistance training could result in the accumulation of metabolites and the inability to perform at high force repeatedly (3). It was reported that resistance training with higher total amount of volume load enhanced both the acute anabolic response (39) and long-term muscular adaptations (40) to resistance training. Therefore, it is suggested that static stretching and walking during inter-set rest intervals of resistance training did not enhance the recovery from muscle fatigue or change the effectiveness of resistance training. Static stretching and aerobic exercise (41) increase muscle blood flow (14), which may facilitate the removal of metabolites. Lopes et al. (41) examined the effects of static stretching (6 types of stretching, 90 s each) before an incremental cycle ergometer test and reported decreased accumulation of lactic acid after the exercise. These data indicated that static stretching before exercise decreases the accumulation of metabolites such as lactic acid. In the present study, there was no significant difference in muscle hardness between interventions, which may indicate that the accumulation of metabolites did not differ between interventions. To our best knowledge, no previous study has examined any effective interventions of inter-set rest intervals in recovery from muscle fatigue. The results of this study suggested that static stretching and walking during inter-set rest intervals of resistance training did not change the number of repetitions to exhaustion or total amount of volume load in non-trained individuals.

There are some limitations in this study. First, this study was conducted on healthy adult males with no previous training experience. Effective rest intervals for resistance training vary depending on the participant's training experience and gender (5, 42). Therefore, the effects of static stretching and aerobic exercise on subjects with different characteristics need to be investigated. Second, metabolites such as lactate acid were not evaluated in this study. Monitoring of these kinetics during resistance training and rest intervals is needed to establish effective recovery methods. Finally, previous studies reported that the effects of static stretching (27, 29, 30) and aerobic exercise (14) were influenced by their duration and intensity. This study used 20 s of static stretching at the intensity of no pain and walking at optimal speed. However, it is possible that higher-intensity interventions were necessary to remove accumulated metabolites more efficiently and recover the fatigue of the quadriceps. In this study, the intensity of aerobic exercise and static stretching were performed at the speed at which participants felt most comfortable and without pain or discomfort, respectively. However, these were based on the subjective sense of the participants, and research using objective intensities is required. This study examined the number of repetitions to exhaustion of knee extension exercises with a load of 70% of maximum strength with 1 min of inter-set rest intervals in untrained participants. The results showed that some participants performed more than 70 knee extension exercises. To our best knowledge, no previous study has examined the number of repetitions of a load of 70% of maximum strength with 1 min of inter-set rest intervals. Therefore, we cannot compare the results of this study with those of previous studies.

In conclusion, this study showed that ROM and muscle hardness increased regardless of static stretching or walking interventions during inter-set rest intervals. In addition, these interventions did not change the number of repetitions to exhaustion or the total amount of volume load of the leg extension exercises. It was suggested that these interventions may not be useful for recovery methods of non-trained individuals.

## Data Availability

The raw data supporting the conclusions of this article will be made available by the authors, without undue reservation.
